# Can dairy help solve the malnutrition crisis in developing countries? An economic analysis

**DOI:** 10.1093/af/vfac083

**Published:** 2023-02-23

**Authors:** Derek Headey

**Affiliations:** The International Food Policy Research Institute, Colombo, Sri Lanka

**Implications**

- Dairy is a high-potential food for addressing child malnutrition in low and middle income countries (LMICs), but consumption varies greatly across LMICs.
- I explore economic drivers of cross-country disparities in dairy consumption among young children.
- Wealth differences across LMICs are the strongest predictor of variation in dairy consumption, followed by dairy price differences.
- Refrigeration also predicts dairy consumption while water quality influences powdered milk consumption.
- LMICs should better leverage dairy policies to improve nutrition, focusing on dairy value chain constraints, industrial policy, trade policy and nutrition education and awareness campaigns.

## Introduction

Agricultural policies are increasingly being asked to do more to address the extensive global burden of undernutrition (Ruel and Alderman, 2013). Undernutrition in early childhood is particularly costly because of its lifelong consequences: poor health, inferior educational outcomes, and lower wages and productivity in adulthood (Black et al., 2013). But to be effective, nutrition-smart agricultural interventions need to produce meaningful dietary improvements very early in life when economically disadvantaged infants and young children are exposed to rising nutrient requirements that are not met by adequate nutrient intake, absorption, and utilization. The intake problem stems from low-quality diets and poor feeding practices, while absorption and utilization problems arise from repeated as well as chronic infections, particularly of the gut.

One area within agriculture with tremendous potential to influence early childhood nutrition is the dairy sector. Dairy products have a range of nutritional and physical characteristics that make them an almost ideal complementary food. Undernourished children in poor countries are often deficient in foods rich in high-quality proteins comprised of essential amino acids that constitute the building blocks for linear growth and cognitive development (Semba, 2016). Dairy has a higher digestibility-corrected amino acid score than any other food (1.21) and is particularly efficacious at closing amino acid gaps in the monotonous diets prevalent in Africa and Asia (FAO, 2013), and in poorer populations more exposed to infections (Semba, 2016). Dairy is unique in stimulating plasma insulin-like growth factor 1 (IGF-1), a growth hormone that acts to increase the uptake of amino acids (FAO, 2013). Dairy is also dense in calories, fat, and various micronutrients (vitamin A and B12), as well as being exceptionally rich in calcium (which contributes to bone length and strength), potassium, magnesium, and phosphorus (Dror and Allen, 2014). Finally, the sheer density of multiple macro- and micronutrients in dairy products—as well as their taste, and familiar texture and consistency—makes them almost ideal for infants and young children with small stomachs incapable of consuming large quantities of nutrient-sparse foods so common in diets of poorer households.

Consistent with the biological importance of milk for nutrition, a diverse and growing body of evidence links dairy consumption to faster growth in early childhood. A public health-nutrition literature has engaged in efficacy and programmatic trials of dairy products on growth in different stages of childhood, and across diverse populations. It finds significant impacts of dairy on child growth (de Beer, 2012; Dror and Allen, 2014). An extensive literature from economic history argues that production of milk—as well as genetic markers of lactose tolerance (Grasgruber et al., 2014)—explains differences in adult height across countries and ethnic groups. In agricultural economics, a recent literature explores the associations between household dairy production, children’s dairy consumption and their linear growth (Rawlins et al., 2014; Hoddinott et al., 2015; Kabunga et al., 2017; Choudhury and Headey, 2018). These studies find strong associations: young children in cattle-owning or dairy-producing households are typically 0.3 to 0.5 standard deviations taller than children from nondairy households. Finally, an extensive analysis of animal-sourced food (ASF) consumption patterns and their associations with stunting among 130,432 children aged 6 to 23 mo from 49 countries finds strong associations between ASF consumption and child growth, particularly for dairy (Headey et al., 2018). Consuming at least two ASFs per day predicted a 5.7-point reduction in stunting, and among different ASFs dairy had significantly stronger negative associations with stunting than meat or eggs.

Yet despite their nutritional potential, dairy consumption in developing regions is highly variable and often very low, as I demonstrate below. Many parts of the developing world have little or no history of dairy consumption, especially in South-East Asia and much of sub-Saharan Africa where lactose intolerance in the adult population is widespread (Silanikove et al., 2015; Anguita-Ruiz et al., 2020). Nevertheless, the experiences of several rapidly transforming Asian economies suggest that dairy consumption can increase dramatically amongst populations without dairy traditions and where lactose intolerance in the adult population is almost universal. In Vietnam, for example, UN surveys indicate that the share of children consuming cow’s milk daily increased from 21% in 2000 to 71% in 2014 (UNICEF 2018). These dairy transformation success stories have relied on domestic production, but also heavily depend on imports to satisfy the growing demand (Headey et al., 2018). This raises an important economic puzzle: why don’t other low-consumption countries import and consume more dairy products? And why don’t policymakers in those countries more aggressively promote dairy consumption and improved supply?

In this paper, I explore why dairy consumption is still low in so much of the developing world where malnutrition is most prevalent, and in spite of abundant evidence of dairy being highly effective in redressing child stunting in particular. Previous reviews on dairy and nutrition in developing countries focus principally on the nutritional properties of dairy (de Beer, 2012; FAO, 2013; Dror and Allen, 2014), the efficacy of dairy interventions (Iannotti, et al. 2013) or environmental sustainability dimensions (Adesogan et al., 2020). Here, I take the nutritional value of dairy as given, and instead focus on economic barriers to increasing dairy consumption in poorer countries. I empirically examine the contributions of poverty and high dairy prices, but also more subtle problems of poor water quality, lack of refrigeration, and cattle ownership. This study does not address other barriers, like issues of lactose intolerance or poor nutritional knowledge on the nutritional benefits of milk, but I conjecture that these are not major barriers. If they were, then the aforementioned dairy success stories from East and South-East Asia are difficult to explain as they have weak dairy traditions and high levels of lactose intolerance. Instead, I aim to demonstrate that economic barriers are major constraints in low income countries, but also that there are important policy levers for making dairy more affordable and accessible to the poor.

## Why Does Dairy Consumption Among Young Children Vary So Much Across the Developing World?

Since malnutrition is especially harmful in early childhood, this section explores the potential determinants of variation in child dairy consumption, as measured by a simple yes/no indicator of whether children 12 to 23 mo consumed milk, yoghurt, or cheese in the past 24 hours as measured by Demographic Health Surveys (DHS) in 59 LMICs (ICF International, 2022) in the 2008 to –2016 timeframe. I first use these cross-country data to describe child consumption differences across countries (Table 1), and then run regressions to explain cross-country differences (Table 2) and assess how well economic factors explain these differences (Table 3). Next, the study turns to an analysis of predictors of dairy consumption in a large DHS sample of 114,560 children in 53 countries to assess which factors explain differences in dairy consumption within countries as opposed to across countries. Our concluding section reflects on the implication of these findings for overcoming economic barriers to greater dairy consumption in the developing world.

**Table 1.** Child dairy consumption and potential predictors of dairy consumption, by region (population-weighted means)

				Ratio of milk price to cheapest staple cereal price (per calorie)							
	*N*	Children consuming dairy in past 24 hours (%)^a^	GDP per capita (2011 PPP$)^b^	Fresh pasteurized milk^c^	Long-life milk^c^	Wealth index (%)	9+ yr maternal education (% mothers)	Born in health facility (% children)	Piped water (% HHs)	Fridge ownership (% HHs)	Rural cattle ownership (%)^a^
Central Africa	7	15.7%	1,670	20.1	5.5	16.9%	17.8%	63.2%	26.8%	6.0%	9.9%
Southern Africa	9	18.0%	1,814	14.5	6.4	16.5%	21.3%	64.9%	27.2%	8.6%	26.7%
West Africa	11	25.0%	3,766	14.3	3.6	33.1%	22.9%	49.3%	19.6%	13.3%	30.4%
Eastern Africa	6	34.4%	1,685	12.5	6.0	16.1%	16.3%	56.3%	28.9%	3.3%	53.5%
South-East Asia	3	19.2%	3,725	16.0	3.9	33.8%	24.4%	54.9%	11.0%	11.1%	31.2%
South Asia	4	52.7%	4,357	5.4	2.5	40.7%	37.6%	74.4%	34.6%	24.1%	51.9%
Middle East N. Africa	4	70.9%	8,139	8.7	2.7	55.1%	46.2%	74.4%	74.2%	87.6%	18.3%
E. Europe C. Asia	7	67.1%	9,587	4.3	1.7	57.3%	89.7%	91.6%	71.7%	74.4%	60.5%
L. America Caribbean	10	58.2%	12,451	6.2	2.6	44.5%	46.8%	79.4%	68.7%	44.8%	23.7%

Notes: Means are weighted with national population estimates from the World Bank (2022), as well as the household survey weights from the DHS. See Supplementary Appendix Table A1 for country-specific results.

Source: a. Demographic Health Surveys (ICF International, 2022); b. World Development Indicators (World Bank, 2022); c. Headey and Alderman (2019).

**Table 2.** Cross-country log–log regressions of the prevalence of child dairy consumption in the past 24 hours as a function of GDP per capita, relative milk prices, nutrition knowledge proxies, and piped water access

	(1)	(2)	(3)	(4)
	Income-price model	Full model with income	Wealth-price model	Full model with wealth
GDP per capita	0.473***	0.241**		
	(0.076)	(0.111)		
Fresh milk price	-0.435***	-0.334***	-0.401***	-0.294***
	(0.108)	(0.110)	(0.104)	(0.102)
Rural cattle ownership		0.063		0.038
		(0.052)		(0.048)
9+ yrs maternal education		-0.071		0.042
		(0.094)		(0.084)
Medical facility births		0.023		-0.228
		(0.189)		(0.174)
Piped water ownership		0.175*		0.337***
		(0.099)		(0.094)
Fridge ownership		0.188**		-0.041
		(0.082)		(0.109)
Wealth index (scaled 0-1)			0.772***	0.753***
			(0.113)	(0.205)
Observations (countries)	58	58	58	58
R-squared	0.636	0.721	0.665	0.759

Notes. Standard errors in parentheses. ***P < 0.01, **P < 0.05, *P < 0.10. All variables are logged, such that the coefficients reported can be interpreted as elasticities. Definitions of variables are provided in the text and in the notes to Table 1.

**Table 3.** Cross-country regressions of child milk consumption as a function of GDP per capita, fresh and long-life milk prices, piped water and interactions between milk prices and piped water

	(1)	(2)
	Linear model	Interaction model
GDP per capita	0.562***	0.439***
	(0.088)	(0.088)
Fresh milk price	-0.429***	-0.726
	(0.125)	(0.648)
Long-life milk price	0.141	-2.164**
	(0.183)	(0.972)
Piped water		-0.707*
		(0.406)
Fresh milk price*piped water		0.100
		(0.181)
Long-life milk price*piped water		0.589**
		(0.267)
Observations	55	55
R-squared	0.676	0.767

Notes. Standard errors in parentheses. ***P < 0.01, **P < 0.05, *P < 0.10. All variables are logged, such that the coefficients reported can be interpreted as elasticities. Definitions of variables are provided in the text and in the notes to Table 1.

### Cross-country patterns of child dairy consumption

Table 1 reports patterns in child dairy consumption and its hypothesized drivers, disaggregated by region (country specific data are reported in Supplementary Appendix Table A1). Child dairy consumption is highly varied across the developing world. One can infer that most young children in Latin America (LAC), Europe and Central Asia (ECA), and the Middle East and North Africa (MNA) consume dairy on a daily basis. About half of children in South Asia (SAS) consumed dairy, mainly driven by India and Pakistan (both at 55%), with just one-third of Bangladeshi children consuming dairy. However, in the remaining regions far fewer children consumed dairy in the past 24 hours of their surveys. In the small sample of South-East Asian (SEA) countries (Myanmar, Cambodia, and Timor-Leste) less than 20% of children consumed milk yesterday. Consumption is similarly low in Central Africa and Southern Africa. In West Africa (WAF), including Nigeria (the most populous countries in Africa and one with very high rates of stunting), just 25% of children consumed milk in the previous day. In Eastern Africa (EAF), which contains substantial highland and pastoral populations with longstanding dairy traditions, over one-third of children consume milk daily, although this masks huge variation. Kenya (58.4%) has by far the highest dairy consumption in the region, followed by Ethiopia (33.5%), Tanzania and Uganda (~28%), Rwanda (18.5%), and Burundi (just 5.6%). In Southern Africa (SAF), just 18% of children consumed dairy in the past 24 hours, with little variation across countries.

The remaining indicators in Table 1 offer some clues as to why dairy consumption is so variable across regions. We report a simple household wealth measure that is scaled 0% to 100% on the number of DHS assets a household owns (including improved housing materials, electricity, improved water and toilets, and consumer durables like TVs and vehicles). This index is clearly strongly correlated with dairy consumption. So too is a measure of the relatively caloric price of milk (which captures how expensive milk calories are relative to starchy staple calories), which we discuss in more detail below. Refrigeration may also explain demand for fresh milk, which is highly perishable, while the quality of water (proxied by piped water) could explain parental demand for powdered milk for their children, as food safety is an important issue for reconstituted milk powder. Proxies for nutritional knowledge like maternal education and exposure to medical services could be important—though they are very indirect proxies at best. Finally, as was noted in the introduction, many country studies focus on cattle ownership and milk consumption in rural areas of developing countries, but across countries that relationship looks weak. Cross-country and within-country regression analyses are presented below to more rigorously assess the predictive power of these factors.

### Do income/wealth differences explain child dairy consumption?

Economic studies on the demand for dairy products suggest that consumption rises quite sharply with income, particularly at low levels of income, even in Africa where lactose intolerance is high (Colen et al., 2018). Moreover, a child-level analysis of the DHS found that children’s consumption of dairy products rises sharply with household wealth; again, even in countries where lactose intolerance is common. Do income or wealth differences account for cross-country differences in child dairy consumption? To examine this, I use GDP per capita in 2011 purchasing power parity (PPP) dollars as a measure of income, as well as a household wealth index estimated from the DHS child level data on ownership of eight assets (excluding piped water or fridge ownership, which are discussed below), with common weights across countries, which is rescaled to vary between 0 and 1.^1^ Per capita GDP and the average household wealth index score are highly correlated with each other across countries (Pearson *r* = 0.62, significant below the 1% level), so separate models with GDP and wealth as interchangeable substitutes are reported in Table 2. Regression 1 in Table 2 estimates an elasticity for GDP per capita of 0.473, which falls to 0.24 (but retains its significance) once other socioeconomic characteristics are added to the model in regression 2. Perhaps unsurprisingly, the elasticity for the DHS wealth measure (Regression 3 in Table 2) is substantially higher, at 0.77, and more robust to the addition of other controls.

**Figure 1.**
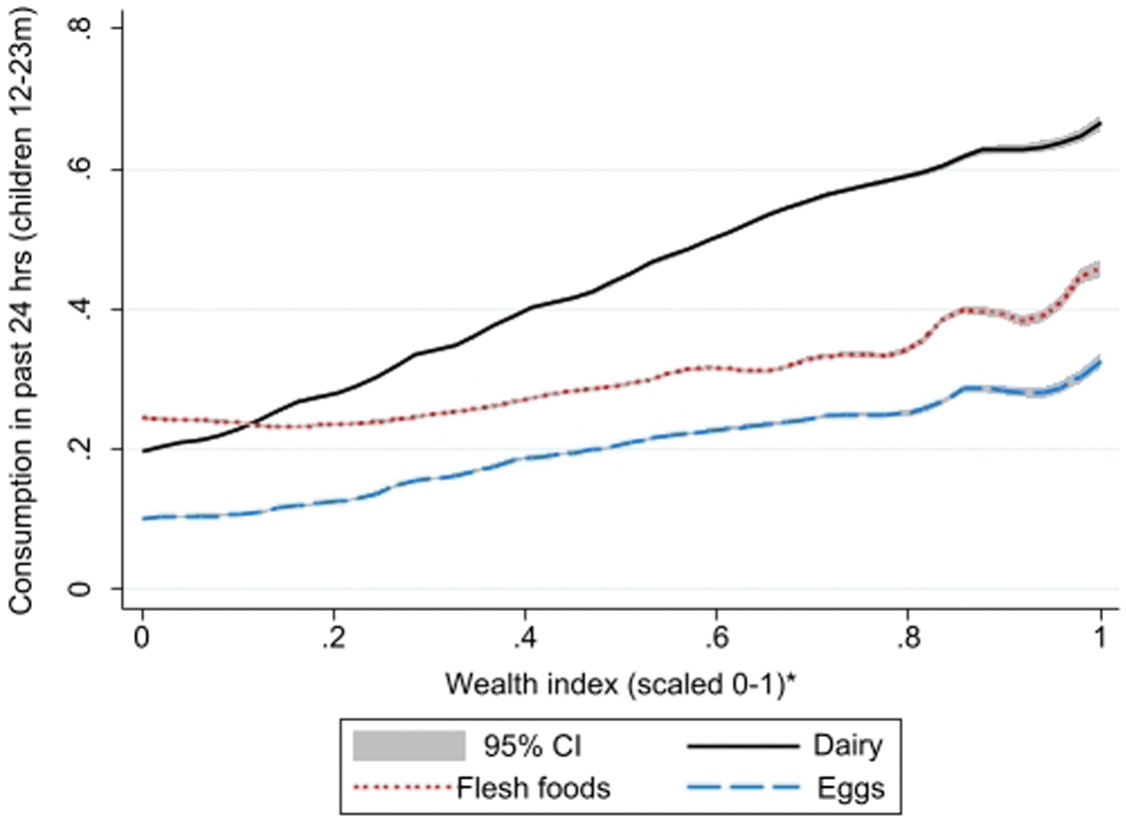
Local polynomial regression estimates with 95% confidence intervals of wealth gradients for children’s consumption of dairy, flesh foods, and eggs in the past 24 hours (114,560 children 12–23 mo of age from 53 developing countries). Source: Authors’ estimates from Demographic Health Survey data (ICF International 2022).

Is wealth also a strong predictor of dairy consumption within countries for the pooled multicountry sample of 114,560 children? Figure 1 reports local polynomial regression estimates of the average relationship between household wealth and children’s consumption of dairy, as well as comparator food groups (eggs and flesh foods). Strikingly, the wealth gradient for dairy is substantially steeper than the gradients for eggs and flesh foods, suggesting that latent demand for dairy consumption among parents of young children is especially strong. This may be because dairy is often viewed as a particularly nutritious food for young children, even among poorly educated populations (Hoddinott, Headey and Dereje, 2015), since it is understood to be a growth-promoting breastmilk.

**Figure 2.**
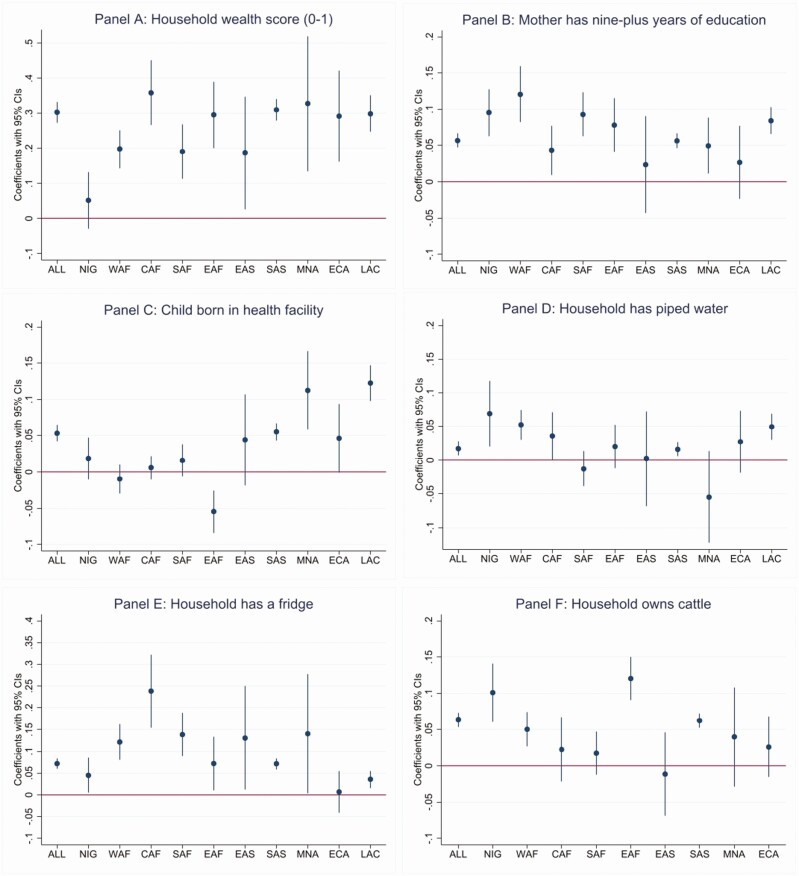
Linear probability model coefficients from region-specific multivariate regressions of dairy consumption in the past 24 hours among children 12–23 mo of age against household wealth, maternal education, access to health facilities, piped water, fridge ownership, and cattle ownership (*N* = 114,560 children). Notes: ALL = All regions; NIG = Nigeria; WAF = West Africa; CAF = Central Africa; SAF = Southern Africa; EAF = Eastern Africa; EAS = East Asia; SAS = South Asia; MNA = Middle East * North Africa; ECA = Eastern Europe and Central Asia; LAC = Latin America Caribbean. Full regressions reported in Supplementary Appendix A.

Figure 1 pools all the child data across countries, but region-specific child-level regressions of dairy consumption in the past 24 hours against a range of household level factors reveal that wealth is generally a strong predictor of dairy consumption (Figure 2, Panel A), with the predicted difference between the richest and poorest children in the sample varying between 18 and 35 percentage points across regions.

### Do retail milk prices explain child dairy consumption?

Poverty is always a constraint to purchasing healthy foods because almost all healthy foods are relatively expensive sources of calories (Headey and Alderman 2019), and poor people must first strive to satisfy calorie requirements before thinking about other health properties of food (or taste). However, for a given level of income, the relative price of a food also matters, as it influences whether households use available income to purchase dairy, for example, or purchase other food groups. Most previous research has not had much to say about price constraints, however, because only recently has comparable international data been available to draw conclusions about cross-country food price differences. Here, I analyze the “relative caloric prices” developed by Headey and Alderman (2019), which measure the prices of milk calories relative to the cheapest staple food calories in each country. These price ratios are currency-free and also capture the cost of diversifying out of starchy staples and into dairy. I examine prices for both fresh pasteurized milk and long-life condensed/powdered milk, which are nationally representative prices collected according to highly standardized definitions of dairy products (see World Bank 2013 and Supplementary Appendix Table A2 for product definitions).

**Figure 3.**
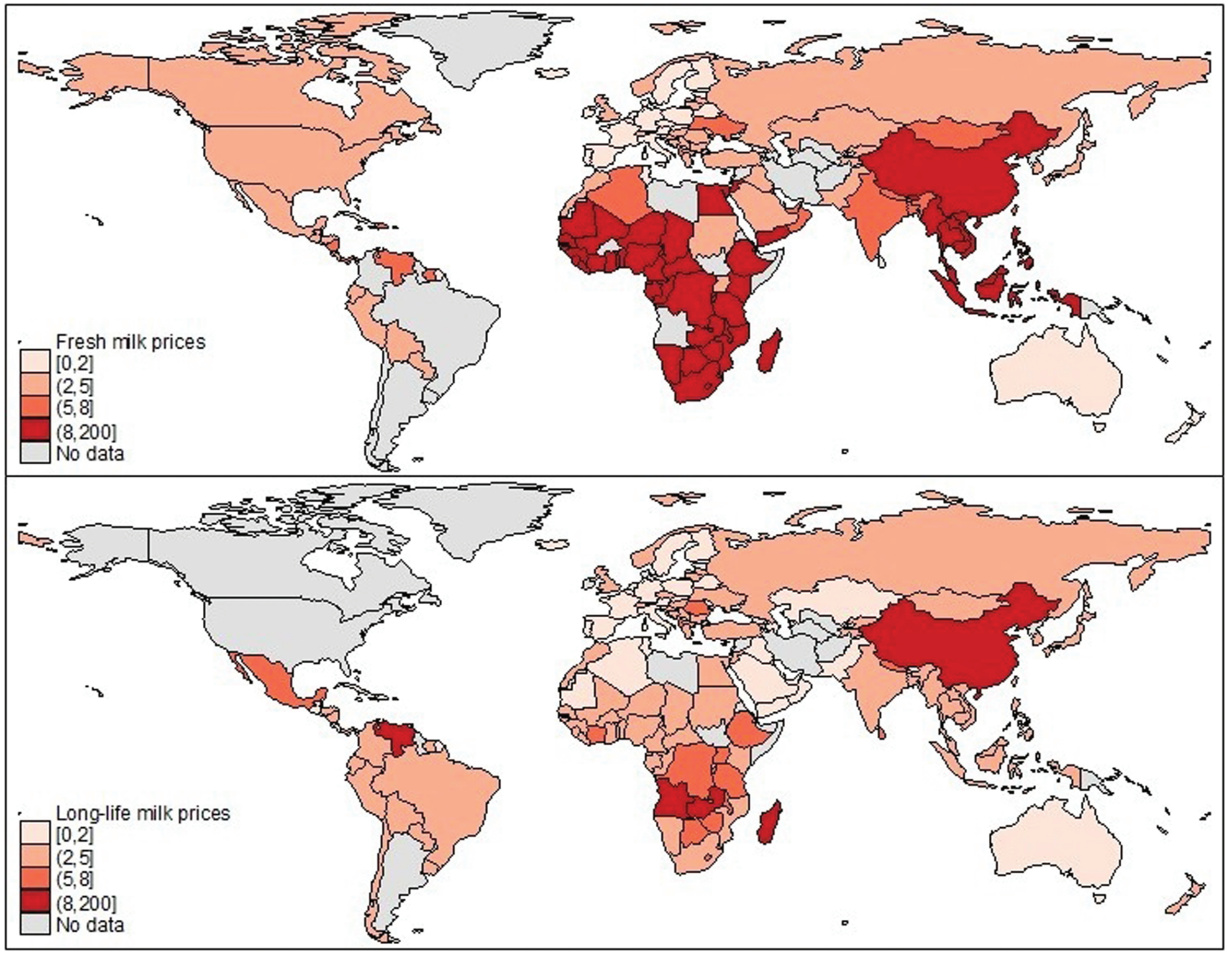
Maps of the ratio of the price of fresh milk and long-life milk calories relative to starchy staple calories in 2011. Source: Author’s construction from the methods described in Headey and Alderman (2019).

Table 1 demonstrated that there are marked differences in the relative caloric prices of both fresh and long-life milk, while Figure 3 presents global maps for both fresh and long-life milk. Fresh milk calories are especially expensive in sub-Saharan Africa and South-East Asia: 20 times more expensive than the cheapest cereal calorie in Central Africa, 12 to 14 times more expensive in other sub-Saharan African regions, and 16 times more expensive in South-East Asia. In contrast, fresh milk calories are relatively cheap in South Asia (5.4 times as expensive as cereal calories) and in most of the more developed regions. Despite the expensiveness of fresh milk, long-life milk (usually powdered) is a relatively cheap source of calories in all regions, though more expensive in sub-Saharan Africa. Hence, one part of the dairy puzzle is why cheaper powdered milk products are not more popular in Africa and South-East Asia.

In Table 2, we observe that these price differences likely account for a substantial share of the difference in the probabilities of child dairy consumption across countries. The estimated elasticity of this probability for the relative price of fresh milk varies between -0.29 and -0.40. Though not reported in Table 2, the estimated elasticity with respect to long-life milk is only -0.18 and not statistically significant at the 10% level (*P*-value = 0.30). Hence, consumption seems more sensitive to the price of fresh milk than to prices of powdered milk, perhaps reflecting parental preferences for fresh milk.

### Does cattle ownership predict greater dairy consumption in rural areas?

A limitation of the consumer price data described above is that the milk prices refer only to marketed retail products, whereas rural households may be much more dependent on milk sold through informal market channels or obtained from a farm household’s own cattle. In the rural Ethiopian highlands, for example, Hoddinott, et al. (2015) found that 90% of the milk consumed by households was produced by those households. To explore the potential important of own-consumption and localized informal markets more, I use a DHS-based indicator based on a simple question of whether the household owns any cattle.

In brief, the cross-country regression evidence in Table 2 suggests cattle ownership does not explain international differences in children’s dairy consumption, but the within-country evidence suggests that children from cattle-owning countries are more likely to consume dairy products than their national peers. Whilst these results seem contradictory, they are actually consistent with other evidence in these results. Because dairy products are tradable, richer populations in Latin America and the Middle East and North Africa, for example, do not need to own cattle to acquire dairy in markets. However, in poorer countries where incomes are low and dairy prices quite high, owning a cow is very advantageous for accessing dairy products. Specifically, the child-level regressions in panel F if Figure 2 shows that cattle ownership increases the probability of consuming dairy by 6 points in South Asia, 5 points in West Africa, 10 points in Nigeria, and 12 points in Eastern Africa. Thus, informal marketing or own consumption of dairy products is clearly important in some parts of the developing world, particularly Eastern Africa where cattle ownership is high, but where dairy processing and formal retailing is less developed than in other regions, such as India.

### Is there any evidence that nutritional knowledge explains child dairy consumption?

Previous research has found strong associations between nutritional knowledge—or its proxies such as formal schooling—and child nutrition outcomes (Webb and Block, 2004). Alderman and Headey (2017) found evidence of significant nutritional benefits to children when mothers had nine or more years of schooling, so I apply this indicator in these regressions also. Nutritional knowledge is also sometimes imparted via exposure to health services, leading us to include an indicator of whether a child was born in a medical facility or not. Both indicators are only very indirect proxies of nutritional knowledge are not specific to dairy knowledge in particular.

There is no evidence that cross-country differences in dairy consumption are explained by these two knowledge proxies. However, child-level regressions reveal that maternal education typically increases the probability of dairy consumption by 4 to 10 percentage points (Figure 2, Panel B), while medical facility births only predict increased consumption outside of sub-Saharan Africa (Figure 2, Panel C). More research is needed on consumer perceptions of dairy’s benefits in the developing world, but I conjecture that poor nutritional knowledge is not generally a binding constraint to increasing dairy consumption. We have already seen that children’s consumption of dairy rises sharply as wealth increases—suggesting that it is a highly preferred food—and it is likely that dairy products are often identified as a nutritious child-friendly food.

### Is water quality a predictor of children’s milk consumption?

In countries that do not produce milk at scale, imported milk powder is often the main alternative. However, since young children are highly sensitive to infection, milk powder requires reconstitution with clean water. Hence, poor access to clean water could add significantly to the implicit cost of milk powder, especially if households—and female caregivers in particular—bear high implicit costs in water collection (Cassivi et al. 2018). I therefore include an indicator of the percentage of households with access to piped water on the grounds that piped water is often more treated for contamination centrally (though not always effectively) and generally piped into the home, which reduces the cost of accessing water. While there is likely to be a strong correlation between household income levels and access to piped water, a key question for us is whether there is some independent impact of water access.

Strikingly, the descriptive statistics in Table 1 shows that piped water access is indeed lowest in those regions where child dairy consumption is also lowest (West and Central Africa and South-East Asia). Moreover, the cross-country regressions in Table 2 suggest that piped water access significantly explains cross-country variation in child dairy consumption even when controlling for income or wealth. The child level regressions in Panel D of Figure 2 suggest some plausible heterogeneity across regions, however: piped water predicts greater consumption of dairy in West and Central Africa and Latin America, three regions where consumption of powdered/condensed milk is relatively high compared to fresh milk.

Given the apparent interaction between dependence on dairy powder imports and water quality, I estimated cross-country regressions that include both fresh milk and long-life milk prices in the same model, before introducing interactions between milk prices and access to piped water (Table 3). Consistent with the inferences above, we observe that the relationship between dairy consumption and long-life milk prices is highly conditional upon access to piped water: caregivers are more sensitive to powdered milk prices in places with better access to piped water.

### Does refrigeration predict more consumption of dairy products?

Given that many consumers may have a strong preference for fresh or long-life liquid milk over powdered milk, refrigeration could be an important magnifier of demand for dairy products (indeed, reconstituted powdered milk also require refrigeration for storage). I therefore use household ownership of a refrigerator (from the DHS survey) as an explanatory variable in both the cross-country and within-country regressions. Fridge ownership is strongly correlated with ownership of other household assets, particularly electricity access, but the goal here is to again test for independent predictive power controlling for household asset ownership.

In the cross-country regressions, we observe mixed evidence on the importance of refrigeration. In the model with GDP per capita as a control (regression 2 in Table 2), the elasticity on refrigeration is moderately large (0.19) and significant at the 5% level, but in the model with household wealth it is highly insignificant, presumably because of its very high Pearson correlation (*r* = 0.86) with the DHS wealth index, which is statistically significant at the 1% level. However, the child level regressions results reported in Panel E of Figure 2 (which also control for household wealth) suggest that fridge ownership is a significant predictor of increased dairy consumption in every region except Eastern ECA (where fridge ownership is very high anyway). The coefficients vary substantially in size, however, and fridge ownership could still partly reflect additional wealth effects rather than a specific impact via cold storage of milk. Even so, it quite self-evident that fridge ownership is almost a prerequisite for consumption of fresh milk–which is highly perishable–but also beneficial for consumption of long-life or powdered milk.

### Using decompositions to account for cross-country differences in child dairy consumption

A notable feature of the cross-country regressions in Table 2 is the explanatory power of international differences in income/wealth and fresh milk prices, and to a lesser extent piped water. In Table 4, I therefore use a simple regression decomposition at means to assess how well the regression models explain differences in children’s dairy consumption between the top ten dairy consumers in the sample and the bottom ten consumers; a massive 61-point difference. In this method, the contribution of each variable to explaining the dairy consumption difference between low and high dairy-consuming countries is simply the product of the difference in that variable (e.g. wealth) and its associated regression coefficient from Table 2 (for a previous example of this kind of approach, see Headey et al. 2015).

**Table 4.** A regression-decomposition of mean differences in child dairy consumption between the top 10 dairy consuming countries and the bottom 10 dairy consuming countries, as defined by the prevalence of dairy consumption in the past 24 hours among children 12–23 mo of age

	Child dairy consumption	GDP per capita	Wealth index (scaled 0–100)	Fresh milk price (relative caloric price ratio)	Piped water access (% of population)
Bottom 10 consumers: means	11.1%	$1,371	19.2%	15.9	23.0%
Top 10 consumers: means	72.5%	$8,326	53.5%	5.9	62.0%
Difference in means	61.4%	$6,955	34.3%	-10.0	39.0%
Difference in logs	0.82	0.78	0.45	-0.43	0.43
Estimated elasticities		0.40	0.71	-0.37	0.26
*Predicted change in dairy consumption (% change)*		*29.3%*	*31.5%*	*16.5%*	*13.2%*
*Predicted change in dairy consumption (% points)*		*23.4 points*	*25.1 points*	*13.2 points*	*10.5 points*
*Share of actual 61.4 point difference in dairy consumption between bottom and top 10 consumers explained by difference in each explanatory variable*		*33.5%*	*36.0%*	*18.9%*	*15.1%*

Notes: These results are based on a linear decomposition at means where income and price elasticities are assumed constant across countries. Elasticities are derived from the regressions in Table 2, but with all variables with insignificant coefficients excluded from the model.

The 61-point difference in dairy consumption prevalence between the low and high-dairy countries is powerfully explained by a simple cross-country model comprised of income/wealth (explaining around 25 points of the 61-point difference), the relative price of fresh milk (13 points) and differences in piped water access (around 10 points). Clearly, long-run economic development (including income growth, greater asset ownership, urbanization and infrastructure development) will eventually help redress the imbalance of dairy consumption observed across countries, but the importance of relative prices arguably offers more scope for increasing consumption quickly through subsidies, trade reform or agricultural investments, as I discuss further below.

## Policy Implications and Areas for  Future Research

This study first identified significant differences in child dairy consumption across regions and showed that these differences are strongly explained by income/wealth differences across countries, as well as wealth differences across households within countries. Although poverty is a lamentable barrier to increasing dairy consumption, the strong wealth gradient for dairy at least suggests that parental demand for their children to consume milk is almost universally strong. However, even for a given per capita income, there are still large variations in dairy consumption across regions, with countries in West and Central Africa and South East Asia (where dairy traditions are weak and cattle ownership not widespread) having particularly low consumption levels compared to similarly poor countries in Eastern Africa or South Asia (with strong dairy traditions).

Given that milk demand rises sharply with income, consumption differences that persist net of income effects likely reflect a combination of formal and informal price differences, with “informal” prices including prices in informal milk markets or the implicit cost of consuming milk that is produced by the household itself. In most of Africa and Asia fresh milk is exceptionally expensive in caloric terms, even in countries where cattle ownership is widespread. This is partially explained by the extreme perishability of fresh milk, and the high transport and trade costs of imported powdered milk, especially in rural areas. However, the low consumption of powdered milk in still somewhat puzzling given that it could surely be consumed on a greater scale in many LMICs, and could also be subsidized for nutritional purposes. Our research hints at water quality as one additional constraint to powdered milk consumption, but one area for future research is better understanding consumer demand for powdered versus fresh milk, as well as demand for nutrient-fortified dairy products targeted at young children.

One plausible conjecture is that in regions where milk is traditionally consumed—such as in East Africa—the preference for fresh milk is stronger than it is for powdered milk. At the same time, many low-income countries at low levels of development have largely failed to modernize their domestic dairy production, collection, processing, storage, transport, and marketing systems (Minten et al. 2020), resulting in most of the milk produced being very vulnerable to spoilage and therefore consumed by the farm households themselves, sold in very poorly developed local markets, or converted into less perishable dairy products (Hoddinott, et al., 2015). However, the history of dairy in India shows that countries at early stages of dairy development can transform their dairy systems rapidly by linking smallholders to larger urban markets—through cooperatives but also private, large-scale commercial firms—and improving both farming and processing and storage technologies (Alderman, 1987).

In countries where milk production is minimal and dairy is not a traditionally consumed good, the challenge of raising dairy consumption is very different to countries with strong dairy traditions like India, Kenya, or Ethiopia. Direct sales of relatively cheap milk powder may not be the most viable way to increase consumption, especially if there is strong and growing demand for liquid milk. In such contexts, one can conjecture that industrial-scale reconstitution of milk, including blending powdered milk with local fresh milk, is a more viable option for improving accessibility and affordability of dairy products. Exemplars in this regard are Thailand and Vietnam. In both countries dairy is not a traditional food, but rising incomes, consumer perceptions of the nutritional importance of milk, and favorable policy environments have led to improved supply and rapid growth in consumption. The broader industrial policy strategies for dairy promotion in these countries is still not well-documented and the existing literature largely focuses on the benefits of these strategies for smallholders, rather than consumers (Morgan, 2009). In Thailand, we know that the incorporation of locally produced milk in school feeding programs was a major source of steady demand for milk producers (Morgan, 2009), and a likely stimulus to consumer awareness of the nutritional importance of milk. In Vietnam, there has been substantial policy support to dairy farmers, but also a favorable business environment for dairy manufacturers. For example, dairy imports face a high tariff (40%) until local supplies are exhausted, at which point tariffs fall to just 5%. This helps domestic dairy farmers, but also ensures manufactures never face supply shortages. Both countries have also seen rapid modernization of milk products and supply chains, including widespread advertising. Learning from these success stories is therefore surely a topic worthy of more research.

The rapid transformation of dairy systems in both traditional and nontraditional countries shows that dairy consumption patterns are not immutable, that there are policy levers for overcoming longstanding constraints to consumption, and that dairy can play a central role in nutrition-sensitive agricultural policies. There is, however, still much to learn about how best to exploit the full potential of dairy for addressing the very extensive and very costly global burden of malnutrition.

## Supplementary Material

vfac083_suppl_Supplementary_Appendix

## Footnotes

1 The assets included in the index and their respective weights are electricity access (0.429), radio (0.057), TV (0.47), refrigerator (0.42), motorbike (0.22), car (0.26), improved flooring (0.39), landline phone (0.22), and mobile phone ownership (0.32). Note that the correlation between this cross-country index and a series of 53 country-specific indices is 0.98, suggesting common weights across countries and a high degree comparability of the index.

## About the Author

**Derek Headey**

**Senior Research Fellow**

International Food Policy Research Institute (IFPRI)

**Derek Headey** is a Senior Research Fellow in the Development Strategies and Governance Division at the International Food Policy Research Institute (IFPRI), where he has worked since 2008. A development economist, his research focuses on the links between agricultural development, poverty and nutrition. He led the *Advancing Research on Nutrition and Agriculture* (**ARENA**) project (2013-2021) for the Bill and Melinda Gates Foundation (BMGF) and worked on agriculture and nutrition projects for USAID in Ethiopia and Myanmar, for BMGF in Burkina Faso, and for UKaid in India, Bangladesh, Nepal, and Pakistan. He has written extensively on the importance of ASFs for nutrition in developing countries, including dairy products. He received his PhD in Economics from the University of Queensland, Australia, in 2008.
