# Supplementary material for: Can dairy help solve the malnutrition crisis in developing countries? An economic analysis
Source: Anim Front. 2023 Feb 23;13(1):7–16. doi: 10.1093/af/vfac083 (PMC9947325; doi:10.1093/af/vfac083)
Supplement: vfac083_suppl_Supplementary_Appendix [file vfac083_suppl_supplementary_appendix.docx]

**Appendix**

**Table A1. Country-specific data on child dairy consumption, GDP per capita, relative prices of fresh and long-life milk**

|  |  | |  | **Ratio of milk price to cheapest staple cereal price (per calorie)** | |
| --- | --- | --- | --- | --- | --- |
|  | **Children consuming dairy in past 24 hrs (%)** | **GDP per capita**  **(2011 PPP$)** | | **Fresh pasteurized milk** | **Long-life (condensed/**  **Powdered) milk** |
| Albania | 91.8% | $10,208 | | 5.8 | NA |
| Armenia | 84.6% | $7,022 | | 4.1 | 2.3 |
| Azerbaijan | 72.2% | $15,754 | | 5.8 | 1.6 |
| Bangladesh | 30.6% | $2,571 | | 7.1 | 3.1 |
| Benin | 33.7% | $1,821 | | 12.5 | 3.3 |
| Bolivia | 56.6% | $5,599 | | 4.3 | 2.5 |
| Brazil | 52.4% | $14,973 | | 7.0 | 2.7 |
| Burkina Faso | 12.6% | $1,473 | | 22.5 | 4.3 |
| Burundi | 5.6% | $772 | | 15.8 | 6.4 |
| Cambodia | 12.3% | $2,659 | | 15.7 | 3.4 |
| Cameroon | 24.5% | $2,969 | | 19.1 | 4.1 |
| Central African | 8.9% | $905 | | 22.2 | 4.5 |
| Chad | 27.0% | $1,864 | | 13.7 | 4.0 |
| Colombia | 72.7% | $11,496 | | 6.1 | 2.2 |
| Comoros | 33.1% | $1,415 | | 17.6 | 4.9 |
| Congo, Dem. Rep. | 7.8% | $682 | | 22.1 | 6.6 |
| Congo, Rep. | 48.5% | $5,214 | | 13.2 | 3.1 |
| Cote d'Ivoire | 14.2% | $2,511 | | 20.7 | 6.0 |
| Dominican Rep. | 83.4% | $11,334 | | 4.7 | 1.7 |
| Egypt, Arab Rep. | 67.8% | $9,824 | | 9.4 | 2.8 |
| Eswatini | 51.3% | $7,324 | | 10.5 | 5.1 |
| Ethiopia | 33.5% | $1,163 | | 8.9 | 6.6 |
| Gabon | 66.0% | $15,894 | | 10.4 | 2.8 |
| Ghana | 21.7% | $3,404 | | 9.6 | 3.4 |
| Guatemala | 46.9% | $6,844 | | 4.0 | 1.3 |
| Guinea | 13.3% | $1,626 | | 13.3 | 5.0 |
| Guyana | 82.4% | $6,131 | | NA | NA |
| Haiti | 24.4% | $1,562 | | 3.2 | 2.9 |
| Honduras | 77.0% | $4,046 | | 3.7 | 3.7 |
| India | 55.2% | $4,636 | | 5.2 | 2.5 |
| Jordan | 90.6% | $9,214 | | 16.8 | 4.8 |
| Kazakhstan | 69.4% | $21,277 | | 3.9 | 1.0 |
| Kenya | 58.0% | $2,557 | | 11.9 | 4.7 |
| Kyrgyz Republic | 60.6% | $2,921 | | 3.7 | 2.1 |
| Lesotho | 35.7% | $2,500 | | 12.2 | 5.8 |
| Liberia | 9.2% | $734 | | 13.3 | 3.5 |
| Madagascar | 25.5% | $1,368 | | 17.7 | 8.1 |
| Malawi | 10.2% | $1,051 | | 8.1 | 5.2 |
| Mali | 34.7% | $1,875 | | 26.8 | 4.7 |
| Morocco | 76.7% | $6,688 | | 4.8 | 2.5 |
| Mozambique | 13.7% | $955 | | 10.3 | 4.4 |
| Myanmar | 21.2% | $3,898 | | 16.0 | 4.0 |
| Namibia | 30.8% | $8,721 | | NA | NA |
| Nepal | 46.7% | $2,031 | | 6.2 | 6.5 |
| Nicaragua | 71.1% | $4,231 | | 4.9 | 4.8 |
| Niger | 18.6% | $802 | | 17.1 | 3.7 |
| Nigeria | 27.5% | $5,259 | | 11.4 | 3.0 |
| Pakistan | 55.1% | $4,310 | | 4.9 | 1.3 |
| Peru | 74.5% | $10,449 | | 4.7 | 3.4 |
| Rwanda | 18.5% | $1,437 | | 11.1 | 4.9 |
| Sao Tome & Principe | 41.3% | $2,696 | | 11.1 | 5.5 |
| Senegal | 42.8% | $2,158 | | 21.2 | 4.8 |
| Sierra Leone | 12.5% | $1,246 | | 17.4 | 4.2 |
| Tajikistan | 70.7% | $2,212 | | 3.4 | 2.4 |
| Tanzania | 28.8% | $2,186 | | 25.4 | 6.5 |
| Timor-Leste | 19.1% | $9,717 | | NA | NA |
| Uganda | 28.2% | $1,603 | | 4.8 | 5.4 |
| Uzbekistan | 60.3% | $4,470 | |  | NA |
| Yemen, Rep. | 68.2% | $3,805 | | 9.0 | 1.8 |
| Zambia | 10.0% | $3,361 | | 22.0 | 9.2 |
| Zimbabwe | 22.8% | $1,667 | | 16.8 | 5.9 |

Sources: a. Demographic Health Surveys (ICF International, 2022); b. World Development Indicators (World Bank, 2022); c. Headey and Alderman (2018).

**Table A2. Definitions of dairy products in the 2011 International Comparison Program**

| **Item name** | **Item description** |
| --- | --- |
| **Fresh pasteurized milk** | |
| Milk, un-skimmed Pasteurized | Number of units: 1: Unit of measurement: Liter: Min: 0.8: Max: 1.5: Brand: Well known: Type: Cow; fresh, un-skimmed milk (whole milk): Packaging: Pre-packed; carton: Processing: Pasteurised; HTST (High Temperature/Short Time) treatment : Fat content: Natural (3-4%): Other features: Not fortified, not flavored: Exclude: UHT (Ultra-high temperature) milk: Comments: Specify brand, weight observed |
| Milk, low-fat, Pasteurized | Number of units: 1: Unit of measurement: Liter: Min: 0.5: Max: 1.5: Brand: Well known: Type: Cow; fresh, low-fat milk: Packaging: Pre-packed; carton: Processing: Pasteurised; HTST (High Temperature/Short Time) treatment : Fat content: Reduced (1-2%): Other features: Not fortified, not flavored: Exclude: UHT (Ultra-high temperature) milk: Comments: Specify brand, weight observed |
| **Long life milk** | |
| Milk, un-skimmed UHT | Number of units: 1: Unit of measurement: Liter: Min: 0.5: Max: 1.5: Brand: Well known/National/Regional Brand: Type: Cow; fresh, un-skimmed milk (whole milk): Packaging: Pre-packed; carton: Processing: Ultra High Temperature (UHT): Fat content: Natural (3-4%): Other features: Not fortified, not flavored : Comments: Specify brand, weight observed |
| Milk, condensed | Number of units: 400: Unit of measurement: Gram: Min: 200: Max: 500: Brand: Well known: Type: Very thick, sweet milk; un-skimmed: Packaging: Pre-packed; tin: Source: Cow; un-skimmed milk (whole milk): Fat content: Approximately 8%: Other features: Sweetened with sugar: Comments: Specify brand, net weight observed |
| Milk, powdered | Number of units: 400: Unit of measurement: Gram: Min: 300: Max: 600: Brand: Well known: Type: Dry powdered milk for adult consumption: Packaging: Pre-packed - Tin or Box: Source: Cow; un-skimmed milk (whole milk) : Comments: Specify brand, net weight observed |

**Table A3. Cross-country correlations between dairy consumption, income, prices and cow ownership for 59 countries**

|  | Child dairy | GDP per capita | Wealth index | Fresh milk price | Long-life milk price | Rural cattle | Maternal education | Medical births | Piped water | Fridge ownership |
| --- | --- | --- | --- | --- | --- | --- | --- | --- | --- | --- |
| Child dairy | 1.00 |  |  |  |  |  |  |  |  |  |
| GDP per capita | 0.63 | 1.00 |  |  |  |  |  |  |  |  |
| Wealth index | 0.83 | 0.62 | 1.00 |  |  |  |  |  |  |  |
| Fresh milk price | -0.61 | -0.43 | -0.47 | 1.00 |  |  |  |  |  |  |
| Long-life milk price | -0.55 | -0.54 | -0.63 | 0.61 | 1.00 |  |  |  |  |  |
| Rural cattle | 0.17 | 0.03 | 0.11 | -0.03 | 0.07 | 1.00 |  |  |  |  |
| Maternal education | 0.65 | 0.63 | 0.69 | -0.47 | -0.46 | 0.16 | 1.00 |  |  |  |
| Medical births | 0.52 | 0.45 | 0.56 | -0.20 | -0.24 | -0.02 | 0.65 | 1.00 |  |  |
| Piped water | 0.64 | 0.58 | 0.59 | -0.41 | -0.35 | 0.10 | 0.58 | 0.63 | 1.00 |  |
| Fridge ownership | 0.82 | 0.68 | 0.86 | -0.48 | -0.54 | 0.07 | 0.74 | 0.57 | 0.64 | 1.00 |

Source: a. Demographic Health Surveys (ICF International, 2022); b. World Development Indicators (World Bank, 2022); c. Headey and Alderman (2018).

**Figure A1. Scatter and LOWESS plots of child dairy consumption against explanatory factors**

**Table A4. Full results from child level regressions of dairy consumption against various household characteristics and controls**

| **Sample>>>** | **ALL** | **NIG** | **WAF** | **CAF** | **SAF** | **EAF** | **EAS** | **SAS** | **MNA** | **ECA** | **LAC** |
| --- | --- | --- | --- | --- | --- | --- | --- | --- | --- | --- | --- |
|  |  |  |  |  |  |  |  |  |  |  |  |
| Household assets (0-1) | 0.30*** | 0.05 | 0.20*** | 0.36*** | 0.19*** | 0.30*** | 0.19** | 0.31*** | 0.33*** | 0.29*** | 0.30*** |
|  | (0.02) | (0.04) | (0.03) | (0.05) | (0.04) | (0.05) | (0.08) | (0.02) | (0.10) | (0.07) | (0.03) |
| 9+ yrs maternal education | 0.06*** | 0.10*** | 0.12*** | 0.04** | 0.09*** | 0.08*** | 0.02 | 0.06*** | 0.05** | 0.03 | 0.08*** |
|  | (0.01) | (0.02) | (0.02) | (0.02) | (0.02) | (0.02) | (0.03) | (0.01) | (0.02) | (0.03) | (0.01) |
| Born in health facility | 0.05*** | 0.02 | -0.01 | 0.01 | 0.02 | -0.05*** | 0.04 | 0.06*** | 0.11*** | 0.05* | 0.12*** |
|  | (0.01) | (0.01) | (0.01) | (0.01) | (0.01) | (0.01) | (0.03) | (0.01) | (0.03) | (0.02) | (0.01) |
| Piped water | 0.02*** | 0.07*** | 0.05*** | 0.04** | -0.01 | 0.02 | 0.00 | 0.02*** | -0.05 | 0.03 | 0.05*** |
|  | (0.01) | (0.02) | (0.01) | (0.02) | (0.01) | (0.02) | (0.04) | (0.01) | (0.03) | (0.02) | (0.01) |
| Household has fridge | 0.07*** | 0.05** | 0.12*** | 0.24*** | 0.14*** | 0.07** | 0.13** | 0.07*** | 0.14** | 0.01 | 0.04*** |
|  | (0.01) | (0.02) | (0.02) | (0.04) | (0.03) | (0.03) | (0.06) | (0.01) | (0.07) | (0.02) | (0.01) |
| Household owns cattle | 0.06*** | 0.10*** | 0.05*** | 0.02 | 0.02 | 0.12*** | -0.01 | 0.06*** | 0.04 | 0.03 | NA |
|  | (0.00) | (0.02) | (0.01) | (0.02) | (0.02) | (0.02) | (0.03) | (0.01) | (0.03) | (0.02) | NA |
| Rural community | -0.02*** | -0.01 | -0.09*** | -0.04** | -0.05*** | -0.05** | -0.04 | -0.02*** | -0.06*** | -0.04 | -0.01 |
|  | (0.01) | (0.02) | (0.01) | (0.01) | (0.02) | (0.02) | (0.04) | (0.01) | (0.02) | (0.03) | (0.01) |
| Child is boy | 0.02*** | -0.01 | -0.01 | 0.00 | -0.00 | 0.03** | 0.02 | 0.02*** | 0.01 | -0.01 | 0.00 |
|  | (0.00) | (0.01) | (0.01) | (0.01) | (0.01) | (0.01) | (0.03) | (0.00) | (0.02) | (0.02) | (0.01) |
| Child Age (months) | 0.00*** | 0.00 | 0.01*** | 0.00 | 0.00 | -0.00*** | 0.01* | 0.00*** | 0.00 | -0.00 | 0.01*** |
|  | (0.00) | (0.00) | (0.00) | (0.00) | (0.00) | (0.00) | (0.00) | (0.00) | (0.00) | (0.00) | (0.00) |
|  |  |  |  |  |  |  |  |  |  |  |  |
| Observations | 114,560 | 5,663 | 10,999 | 6,438 | 7,464 | 8,522 | 2,675 | 51,369 | 4,124 | 3,422 | 18,645 |
| R-squared | 0.06 | 0.03 | 0.12 | 0.23 | 0.13 | 0.13 | 0.06 | 0.05 | 0.05 | 0.21 | 0.13 |

Notes: ALL=All regions; NIG=Nigeria; WAF=West Africa; CAF=Central Africa; SAF=Southern Africa; EAF=Eastern Africa; EAS= East Asia; SAS=South Asia; MNA=Middle East * North Africa; ECA=Eastern Europe & Central Asia; LAC=Latin America Caribbean. *, ** and *** refer to significance at the 10%, 5% and 1% level respectively. Standard errors are clustered at the DHS cluster level. All regressions control for country fixed effects.
